# Supplementary figures and images for: A Pervasive History of Gene Flow in Madagascar’s True Lemurs (Genus Eulemur)
Source: Genes (Basel). 2023 May 23;14(6):1130. doi: 10.3390/genes14061130 (PMC10298339; doi:10.3390/genes14061130)

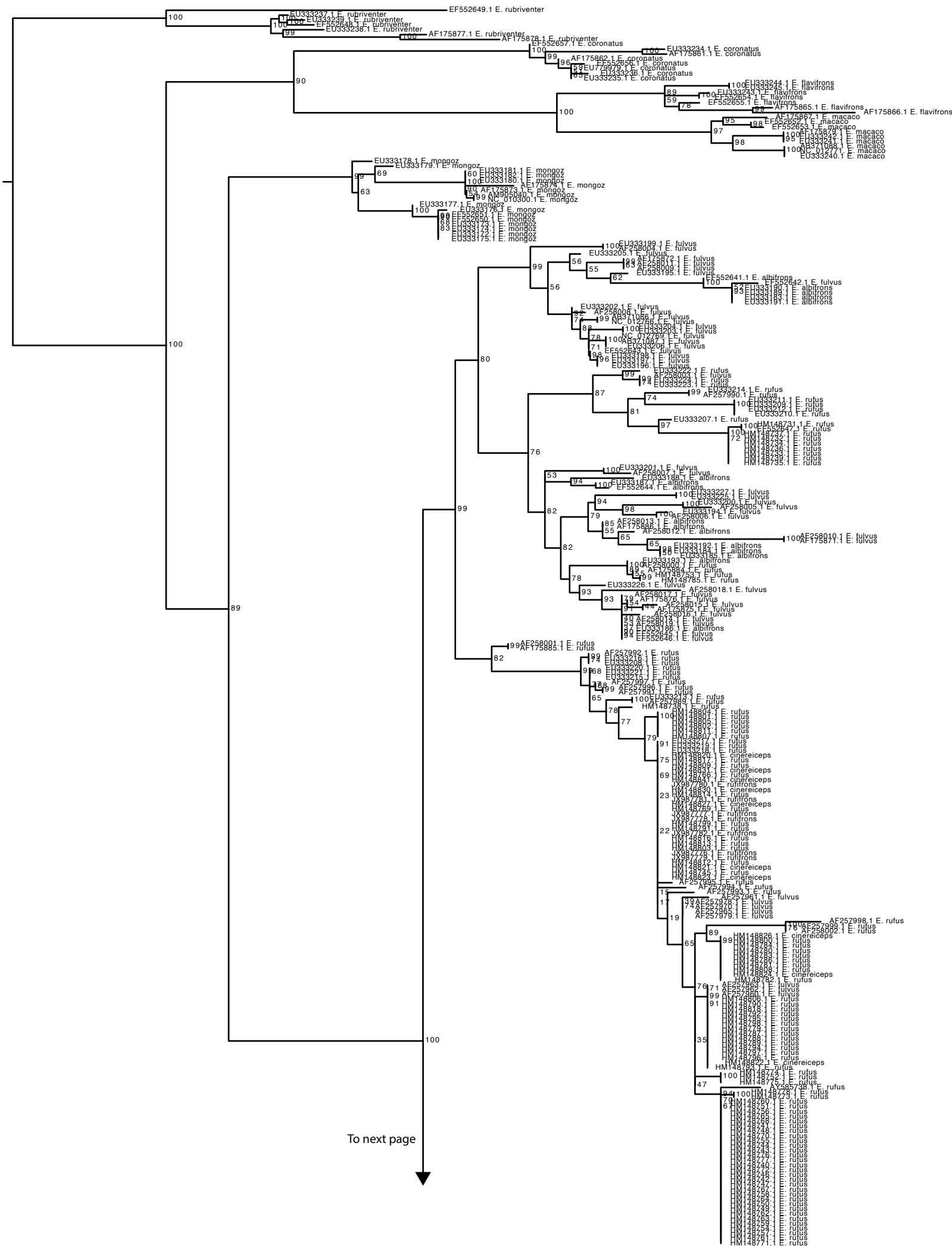

To next page

Supplement: Supplementary file 1 [file genes-14-01130-s001.zip › FigS1_DetailedMitochondrialTree.pdf]
